# 

**Published:** 1883-06

**Authors:** 


					﻿Whole Number)
cccCexT
June, 1883.
Number 6,
Vol. XLVI..
THE
C H ICAGO
MEDICAL
Journal & Examiner.
CHICAGO:
PUBLISHED BY THE CHICAGO MEDICAL PRESS ASSOCIATION
188 Clark Street.
^‘Read the Notice of this Journal among Advertisements.
				

